# Endocrine and enzymatic shifts during insect diapause: a review of regulatory mechanisms

**DOI:** 10.3389/fphys.2025.1544198

**Published:** 2025-03-14

**Authors:** Hamzeh Izadi

**Affiliations:** Department of Plant Protection, Faculty of Agriculture, Vali-e-Asr University of Rafsanjan, Rafsanjan, Iran

**Keywords:** diapause, enzymes, hormones, cold tolerance, cryophysiology, dormancy

## Abstract

Insect diapause is a vital survival strategy that enables insects to enter a state of suspended development, allowing them to withstand unfavorable environmental conditions. During diapause, insects significantly lower their metabolic rate and build up energy reserves, which they gradually utilize throughout this period. The regulation of diapause involves a complex interaction of hormones and enzymes. Juvenile hormones (JHs) affect adults and larvae differently; in adults, the absence of JH typically triggers diapause, while in larvae, the presence of JH encourages this state. Ecdysteroids, which regulate molting and metamorphosis, are carefully controlled to prevent premature development. Reduced signaling of insulin-like peptides enhances stress resistance and promotes energy storage. Several enzymes play crucial roles in the metabolic adjustments necessary for diapause. These adjustments include the degradation of JH, the ecdysteroidogenic pathway, and the metabolism of fatty acids, glycogen, cryoprotectants, and stress responses. Understanding diapause’s molecular and biochemical mechanisms is essential for fundamental entomological research and practical applications. Despite recent advances, many aspects of diapause regulation, especially the interactions among hormonal pathways and the role of enzymes, remain poorly understood. This review analyzes approximately 250 papers to consolidate current knowledge on the enzymatic and hormonal regulation of diapause. It offers a comprehensive overview of key processes based on recent studies and suggests future research directions to fill gaps in our understanding of this significant biological phenomenon. The review also lays the groundwork for enhancing pest control strategies and ecological conservation by deepening our understanding of diapause mechanisms.

## Introduction

### Diapause

Insects have developed various survival strategies to cope with unfavorable environmental conditions, with diapause being one of the most remarkable (Denlinger, 2023). Diapause is a developmental arrest involving significant metabolic suppression. This adaptive trait is ecologically important as it synchronizes the insect’s life cycles with optimal environmental conditions, playing a crucial role in species distribution and evolution (Hand et al., 2016; Kaur Gill et al., 2017; Tougeron, 2019). A complex interaction of genetic, hormonal, and enzymatic mechanisms controls the regulation of diapause. Environmental signals, such as photoperiod and temperature, act as primary cues for initiating diapause. These signals are translated into physiological responses through hormonal systems, including juvenile hormones (JHs), ecdysteroids, and insulin-like peptides (ILPs). Enzymatic pathways and hormonal signals are intricately interconnected during insect diapause, collaboratively regulating metabolic suppression, enhancing stress resistance, and arresting development. Hormones serve as master regulators, with enzymatic pathways acting as effectors to precisely initiate, maintain, and terminate diapause. Additionally, dynamic feedback loops and crosstalk between hormonal signals and enzymatic activities tightly coordinate this process. The delicate balance between hormonal modulation and enzymatic regulation is critical for diapause survival and successful post-diapause reactivation (Kaur Gill et al., 2017; Roncalli et al., 2021; Lebenzon et al., 2022; Denlinger, 2023). Key enzymatic pathways involved in diapause include lipid metabolism, glycogen storage, and the synthesis of cryoprotectants such as trehalose, glycerol, and sorbitol. These molecules prevent cellular damage during prolonged periods of environmental stress, such as freezing or desiccation. Additionally, antioxidant enzymes play a vital role in neutralizing reactive oxygen species (ROS) generated during metabolic suppression, safeguarding cellular integrity (Weber et al., 1967; Shinoda and Itoyama, 2003; Tollarova, 2008; Niwa and Niwa, 2014; Denlinger, 2023). Despite decades of research, many aspects of the molecular and biochemical regulation of diapause remain poorly understood. Existing studies often focus on specific species or limited aspects of diapause regulation, leaving gaps in our understanding of how hormonal and enzymatic pathways interact to maintain diapause and respond to environmental changes. This knowledge is not only theoretically important but also has practical implications. Insights into the mechanisms of diapause can enhance pest management strategies by predicting periods of insect vulnerability and improving the overwintering success of beneficial insects used in biological control. This review brings together current knowledge on hormonal and enzymatic regulation of diapause, focusing on their roles in metabolic adjustment, stress tolerance, and developmental arrest. By integrating recent findings, the manuscript aims to identify key gaps in understanding and propose future directions for research in this rapidly evolving field.

### Diapause and insect’s developmental stage

**FIGURE 1:**
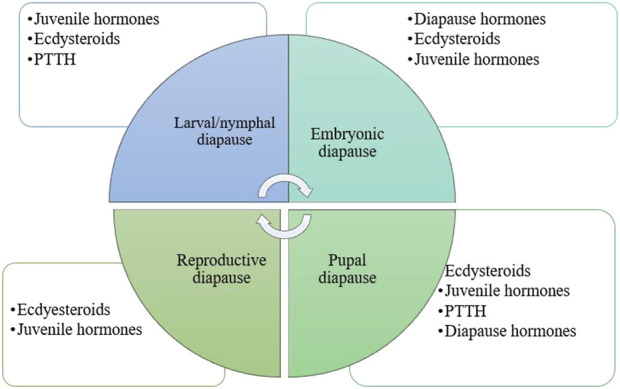
Diapause and the hormones are involved in various developmental stages of insects. Hormones play a crucial role in insect diapause, with their specific types and significance varying by species and the developmental stage of the insect during this process. PTTH, prothoracicotropic hormone (Kaur Gill et al., 2017; Karp, 2021; Denlinger, 2022; Denlinger, 2023).

Diapause can occur at any developmental stage of insects (Figure 1). Embryonic diapause is a phase of halted development in embryogenesis that many insect species use to synchronize egg hatching with the most favorable conditions. A dramatic reduction or complete cessation of mitotic activity characterizes the process. Embryonic diapause can happen during any stage of embryonic development (Karp, 2021). Reproductive diapause is a cessation of reproduction that marks adult diapause (Hodek, 2012). It is a prevalent photoperiodic regulation in insects, with both males and females entering diapause in many species. During diapause, females have small ovaries with oocytes containing little or no yolk, while the development of the male accessory glands is typically suppressed. Mating behavior is largely suppressed during diapause, with many species typically mating in the spring after diapause ends (Nadeau et al., 2022). Hormonal regulation of adult diapause entails an intricate interaction of multiple hormones, particularly ecdysteroids and juvenile hormones (Ma et al., 2021a; 2021b). In addition to hormones, various other factors such as energy sensing, stress response, insulin-like signaling, and the TOR pathway can also affect the reproductive diapause of insects (Eustice et al., 2022). During immature stages, diapause significantly slows or stops development. This type of diapause may occur during the larval, nymphal, or pupal stages. Larval diapause is frequently observed in the life cycles of holometabolous insects, especially in lepidopteran, coleopteran, hymenopteran, and some dipteran species (Dittmer and Brucker, 2021). Nymphal diapause is well-studied in hemipteran species (Zhai et al., 2017). During pupal diapause, there are strong gene expression dynamics, revealing a preprogrammed transcriptional landscape that is active in the winter (Pruisscher et al., 2022).

### Ecophysiological phases of diapause

**FIGURE 2:**
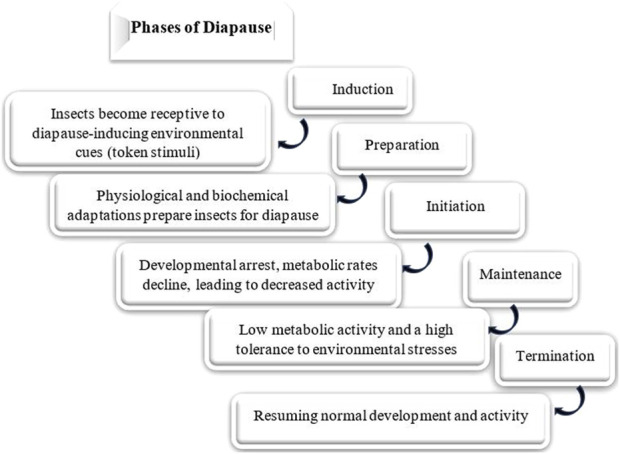
Phases of insect diapause: the progression through these phases is influenced by both genetic and environmental factors. The specific timing and duration of each phase can vary among species, populations, and even individuals (Koštál, 2006; Kaur Gill et al., 2017; Tougeron, 2019; Cambron-Kopco et al., 2022).

**FIGURE 3:**
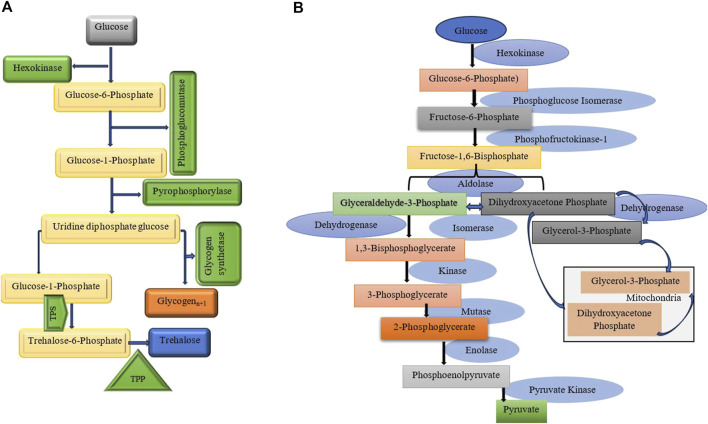
**(A)** The key aspects of glycogen and trehalose metabolism, their connections, and the enzymes involved. Trehalose synthesis and cryoprotectant production pathways are highlighted. It involves two key enzymes: Trehalose-6-phosphate synthase (TPS): Which catalyzes the formation of trehalose-6-phosphate from glucose-6-phosphate and UDP-glucose. Trehalose-6-phosphate phosphatase (TPP): Converts trehalose-6-phosphate into trehalose by removing the phosphate group (Silljé et al., 1999; Badaruddin et al., 2013; Seo et al., 2018). **(B)** This diagram illustrates glycolysis, a vital metabolic pathway that converts glucose into pyruvate, producing ATP. The process includes ten enzymatic steps, regulated by three key enzymes that manage its flow (Kunieda et al., 2006; Harris and Harper, 2015).

**FIGURE 4:**
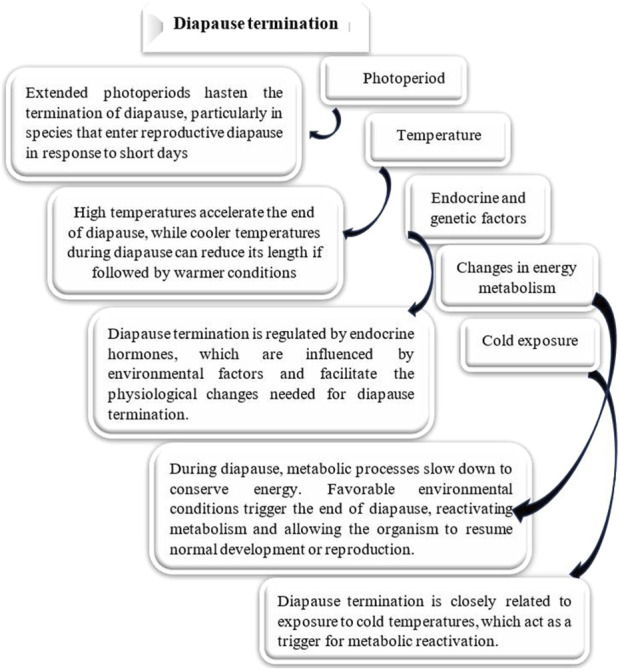
Effect of different factors on diapause termination. Various environmental factors significantly influence Diapause termination in insects, affecting both the duration and intensity of this developmental pause (Chippendale, 1977; Hand et al., 2016; Kaur Gill et al., 2017; Tougeron, 2019; Denlinger, 2023).

Diapause encompasses several ecophysiological phases: induction, preparation, initiation, maintenance, termination, and post-diapause, all regulated by exogenous and endogenous factors. These phases are marked by shifts in metabolic rates and physiological adaptations (Figure 2) (Koštál, 2006). The process of entering diapause begins with an insect responding to environmental signals, such as photoperiod, temperature, and food availability. This phase involves halting morphological development, changes in coloration or behavior, and secretion of specialized enzymes or hormones. Nutrient reserves, including lipids, proteins, and carbohydrates, are accumulated to sustain energy demands during diapause. Moreover, metabolism shifts from energy-intensive growth to glycolysis and gluconeogenesis, helping conserve energy (Figures 3A,B) (Cambron-Kopco et al., 2022; Denlinger, 2023; Dong et al., 2019; Han and Bauce, 1998; Koštál, 2006; Koštál et al., 2017). The physiological mechanisms regulating nutrient homeostasis during diapause include pathways such as insulin signaling, AMP-activated protein kinase, and adipokinetic hormones (Hahn and Denlinger, 2007). While the biochemical pathways remain incompletely understood, nutrient storage directly influences diapause onset, duration, and post-diapause fitness (Batz and Armbruster, 2018; Short and Hahn, 2023). During diapause maintenance, insects experience metabolic depression and developmental arrest, relying on stored energy reserves, primarily lipids (Lehmann et al., 2016). Sensitivity to environmental cues increases as this phase progresses, signaling the approach of diapause termination (Koštál, 2006). Energy conservation is critical, with metabolic resources carefully managed to ensure survival and support post-diapause development. The use of stored nutrients, especially glycogen, also facilitates cryoprotectant production to mitigate the effects of low temperatures (Hahn and Denlinger, 2010). Genetic factors, metabolic processes, and environmental conditions influence diapause termination. However, it is often simply a consequence of the passage of time (Figure 4) (Robbins et al., 2010; Ragland et al., 2019; Poitou et al., 2020). The successful transition out of diapause depends on sufficient energy reserves to fuel post-diapause recovery and growth. The precise timing of diapause termination is key to optimizing survival and distribution (Arrese and Soulages, 2010; Hahn and Denlinger, 2010; 2007; Xu et al., 2021). Diapause termination in insects involves shifts in energy metabolism. They move from a state of metabolic depression to increased respiratory activity, which helps meet the higher energy demands for movement, feeding, and reproduction (Jiang et al., 2010; Dong et al., 2019; Karp, 2021). These changes involve elevated metabolic rates regulated by various hormones and enzymes that control energy production and utilization. Additionally, diapause termination is linked to resource allocation and morphogenesis, underscoring the importance of metabolic regulation during this phase. Proper coordination of these processes ensures a successful transition from dormancy to full physiological activity, allowing the insect to re-enter its life cycle and meet ecological challenges effectively (Jiang et al., 2010; Dong et al., 2019; Karp, 2021).

### Enzyme activity during diapause

**FIGURE 5:**
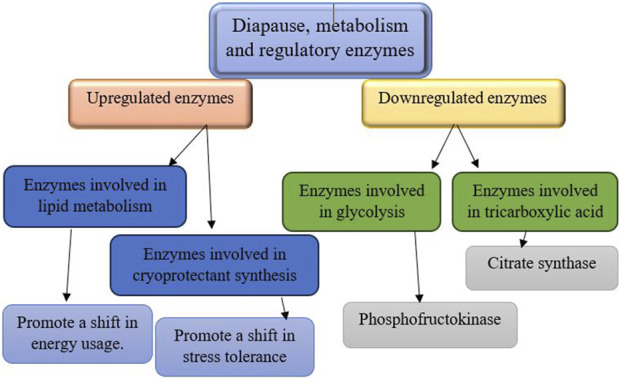
The regulation of metabolic enzymes during diapause involves downregulation and upregulation processes (Hahn and Denlinger, 2010; Uzelac et al., 2020; Huang et al., 2022).

**FIGURE 6:**
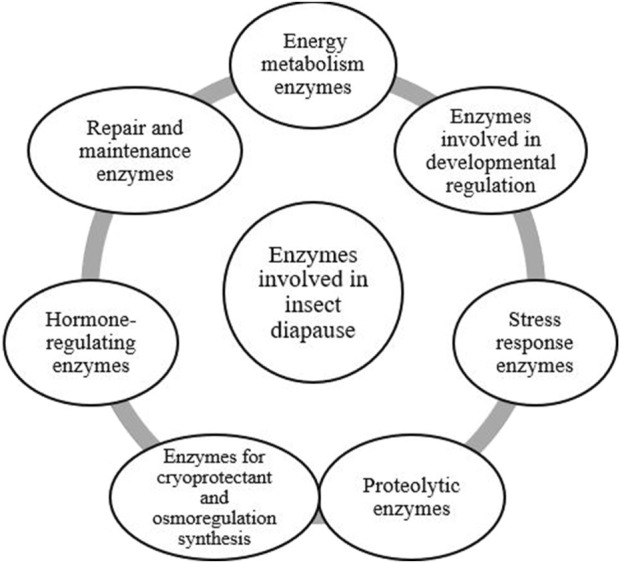
The classification of enzymes involved in insect diapause, organized according to their primary functions (Shappirio, 1974; Joanisse and Storey, 1995; Tollarova, 2008; Mohammadzadeh and Izadi, 2016; Mohammadzadeh and Izadi, 2018; Uzelac et al., 2020).

The biochemical processes that enable insect diapause represent a sophisticated strategy that underscores the complexity of diapause and offers insights into its broader ecological and evolutionary significance (Hahn and Denlinger, 2010). Enzymes, proteins that serve as catalysts, play a vital role in maintaining cellular processes and functions (Piumetti and Illanes, 2022). During diapause, the activity of certain enzymes including those involved in glycogen and lipid metabolism, and protein synthesis, is known to be altered to support the metabolic slowdown (Figures 5, 6) (Hahn and Denlinger, 2010). Some enzymes, especially those related to stress responses, remain active during diapause, helping insects survive harsh conditions. The specific enzymes involved in insect diapause vary depending on the insect species and their diapause strategy. Enzymes that play a role in insect diapause can be classified according to their activity and the biological processes they regulate (Table 1). These enzymes facilitate metabolic adjustments, energy conservation, stress resistance, and other physiological changes essential for diapause. This review focuses on enzymatic pathways related to hormonal regulation, glycogen metabolism, cryoprotectant synthesis, and oxidative stress during diapause.

**TABLE 1:** The classification of enzymes involved in insect diapause is organized according to their primary functions.

Main category	Primary function	Subcategory	Function	Examples
Energy metabolism enzymes	Regulation of the shift in energy production and storage during diapause	Glycolytic enzymes	Breakdown of glucose	Hexokinase, phosphofructokinase
		Gluconeogenic enzymes	Glucose synthesis	Glucose-6-phosphatase, fructose-1,6-bisphosphatase
		Lipid metabolism enzymes	Mobilizing and storing lipids	Lipase, Acyl-CoA synthetase
		Oxidative phosphorylation enzymes	Regulate mitochondrial energy production	Cytochrome c oxidase, ATP synthase
Stress response enzymes	Maintenance of cellular integrity under environmental stress during diapause	Antioxidant enzymes	Mitigate oxidative damage caused by reactive oxygen species	Superoxide dismutase, catalase, glutathione peroxidase
		Cryoprotectant-related enzymes	Facilitate the production of cryoprotectants	Aldose reductase (glycerol synthesis), trehalase (trehalose synthesis and breakdown)
		Ion-regulating enzymes	Maintaining ion gradients	Na+/K+-ATPase
Proteolytic enzymes	Regulate protein turnover to manage cellular repair and energy redistribution	Proteases	Break down proteins into amino acids for recycling or energy	Cathepsins, serine proteases
Hormone-regulating enzymes	Control hormonal pathways that regulate diapause	Juvenile hormone regulatory enzymes	Control juvenile hormone levels	Juvenile hormone esterase, juvenile hormone epoxide hydrolase
		Ecdysteroid-related enzymes	Involved in the production or breakdown of ecdysteroids	Ecdysone 20-monooxygenase

#### Hormones, diapause, and regulatory enzymes

The initiation and regulation of diapause are controlled by complex hormonal and enzymatic processes. The production and breakdown of hormones linked to insect diapause require the involvement of multiple enzymes. Understanding these hormonal processes and the related enzymes is crucial for understanding how insects adjust to seasonal changes, and could provide insights into how diapause is initiated and sustained. The primary hormones involved in diapause regulation are prothoracicotropic hormones (PTTHs), juvenile hormones (JHs), diapause hormones (DHs), and ecdysteroids (Renfree and Shaw, 2000; Denlinger, 2002; Hand et al., 2016; Harsimran et al., 2017).

#### Juvenile hormones

**FIGURE 7:**
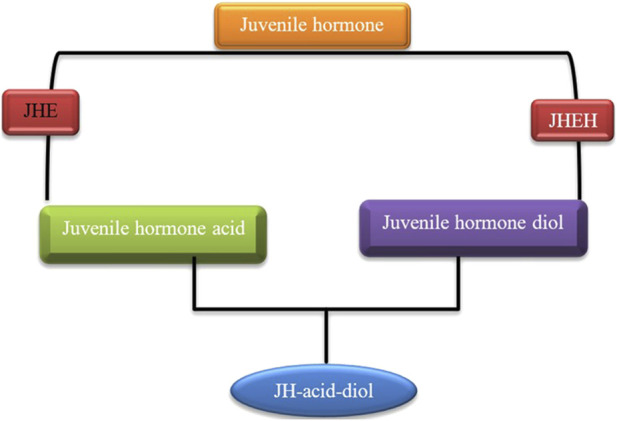
The role of juvenile hormone esterase (JHE) and juvenile hormone epoxide hydrolase (JHEH) in modulating JH levels during insect diapause. Elevated activity of these enzymes triggers reproductive dormancy by lowering JH titers (Noriega et al., 2006; Kamita and Hammock, 2010; Tusun et al., 2017).

Juvenile hormones (JHs) are sesquiterpenoid hormones produced by the corpora allata, critically regulating insect development, reproduction, and diapause. During diapause, JH levels are typically suppressed, leading to the arrest of reproductive and developmental processes, particularly in species undergoing adult diapause (JH level is usually high during larval diapause). This suppression is mediated by the enhanced activity of JH-degrading enzymes, including juvenile hormone esterase (JHE) (Figure 7) (Table 2) and juvenile hormone epoxide (JHEH) hydrolase (Hutfilz, 2022). For example, in *Coccinella septempunctata*, increased JHE and JHEH activity correlates with ovarian dormancy and suppressed reproductive behavior (Li et al., 2022). Similarly, in *Ostrinia nubilalis*, JHE is the key enzyme controlling JH catabolism (Bean et al., 1982), whereas, in *Sesamia nonagrioides*, the decline in JH levels is more dependent on reduced biosynthesis than enzymatic degradation (Mane and Chippendale, 1981), illustrating species-specific regulatory mechanisms. At the molecular level, diapause-associated shifts in JH signaling involve changes in the expression of genes encoding JHE and JHEH. Upregulation of these genes during the early diapause phase supports the maintenance of metabolic suppression and developmental arrest. Notably, silencing these genes disrupts diapause by sustaining elevated JH levels, promoting reproductive processes such as ovarian maturation (Noriega, 2014; Zhang et al., 2022). The data, however, indicate that JHE is not essential for the transition between larval development, diapause, and metamorphosis in the Mediterranean corn borer, *S. nonagrioides* (Schafellner et al., 2008). Juvenile hormone biosynthesis, rather than the JH degradation pathway, may determine the decrease in JH levels in some diapausing insects (Gao et al., 2022). Moreover, JH signaling plays a crucial role in regulating reproductive diapause. This adult diapause mainly occurs when genes responsible for producing JH-binding proteins, JH esterase, JH acid methyltransferase (JHAMT), JH epoxide hydrolase, and fatty acid synthase are suppressed (Ma et al., 2021a). JHAMT is an enzyme found in insects that plays a role in the production of JH. In the final stages of this process, JHAMT transfers a methyl group from a compound called S-adenosyl-L-methionine to either farnesoic acid (FA) or JH acid (JHA) (Li et al., 2013). During the nymphal diapause of *Laodelphax striatellus* (Hemiptera: Delphacidae) the gene JHAMT was upregulated, whereas, the gene cytochrome P_450_ monooxygenase (CYP314A1, Shd) was downregulated (Zhai et al., 2017). During the adult emergence in the Eri silkworm, *Samia cynthia ricini*, two enzymes, 3-hydroxy-3-methylglutaryl CoA reductase, and JHAMT, are involved in the JH synthesis pathway (Sheng et al., 2008). Recent studies have highlighted interactions between JH and other hormonal pathways during diapause. For instance, JH cross-talks with insulin-like peptides (ILPs) and ecdysteroids to coordinate metabolic conservation and stress tolerance (Karpova et al., 2013; Gijbels et al., 2019). Despite advancements, gaps remain in understanding how environmental cues modulate JH signaling, particularly the mechanisms integrating photoperiod and temperature with JH biosynthesis and degradation. Future research should focus on elucidating how environmental and genetic factors collaboratively regulate the JH pathway. Identifying the upstream regulators of JHE and JHEH expression, as well as exploring their interactions with other enzymes in the JH signaling cascade, could provide valuable insights into the initiation and maintenance of diapause. However, this enzymatic suppression of JH offers a target for disrupting diapause in pest species or extending dormancy in beneficial insects.

**TABLE 2:** Enzymes play a role in the metabolism of juvenile hormones during insect diapause.

Regulatory enzyme	Function	References
Juvenile hormone esterases (JHEs)	Metabolizing JH through ester bond hydrolysis	Zhang et al. (2022)
Juvenile hormone epoxide hydrolase	Inactivation of JH through hydrolysis of the epoxide functional group	Tusun et al. (2017)
Juvenile hormone acid methyltransferase	Converts JH acids or inactive precursors to active JH at the final step of the JH biosynthesis pathway	Shinoda and Itoyama (2003)
3-hydroxy-3-methylglutaryl CoA (HMG-CoA) reductase	Catalyzes the conversion of HMG-CoA to mevalonate at the JH biosynthesis pathway	Wang et al. (2023)
Cytochrome P450 monooxygenase	Regulating juvenile hormone biosynthesis	Guo et al. (2024)

#### Diapause hormone (DH)

In *Bombyx mori*, embryonic diapause is intricately regulated by the diapause hormone (DH), a neuropeptide released from the suboesophageal ganglion, which orchestrates the onset, continuation, and termination of the dormant state. Environmental cues trigger shifts in hormonal and enzymatic pathways, with DH playing a central role. Specifically, DH enhances trehalase activity in developing ovaries, leading to a buildup of glycogen in mature eggs, a process known as hyperglycogenism that is essential for initiating diapause (Table 3) (Yamashita, 1996; Denlinger et al., 2012; Jiang et al., 2019; Chen L. et al., 2022). A study found that both diapausing and non-diapausing eggs contain the enzyme esterase A, but this enzyme is inactive in non-diapausing eggs. In diapausing eggs, especially those that have been chilled, there is a surge in esterase A activity right before glycogen begins to reappear. This increase closely correlates with the onset of hatching, indicating that the enzyme plays a crucial role in ending diapause rather than in later developmental stages (Kai and Nishi, 1976). Research into gene expression patterns has revealed that certain enzymes, such as glycogen phosphorylase, phosphofructokinase, sorbitol dehydrogenase-2, and glucose-6-phosphate dehydrogenase, show distinct activity profiles in diapausing versus non-diapausing eggs. For example, the glycogen phosphorylase gene remains continuously active in non-diapause eggs, while in diapause eggs, its activity is high during the early phase but decreases later on. Similar patterns of change are observed with the other metabolic genes, indicating a carefully regulated energy metabolism during diapause (Saravanakumar et al., 2008). Comparative examples further illustrate the diversity of diapause hormone’s role in diapause. In *Helicoverpa* moths, higher levels of DH in non-diapausing pupae rapidly terminate diapause (Zhang et al., 2015), whereas, in *Drosophila melanogaster*, DH stimulates trehalase activity to promote the conversion of glycogen into glycerol and sorbitol, compounds that function as cryoprotectants to safeguard embryos during dormancy (Horie et al., 2000). Together, these enzyme–hormonal interactions underscore the complex molecular mechanisms that ensure the survival of diapausing eggs while allowing development to resume when environmental conditions improve.

**TABLE 3:** Enzymes play a role in the metabolism of diapause hormone during insect (*Bombyx mori*) diapause.

Regulatory enzyme	Function	References
Trehalase	A glycoside hydrolase catalyzes the conversion of trehalose to glucose	Terra et al. (2019)
Esterase A	Hydrolyze the compounds that contain ester, amide, and thioester bonds	Ding et al. (2022)
Glycogen phosphorylase	Catalyzes the initial reaction of glycogen degradation, converting glycogen to glucose-1-phosphate	Livanova et al. (2002)
Phosphofructokinase	In glycolysis, catalyzes the phosphorylation of fructose-6-phosphate	Wegener and Krause (2002)
Glucose-6-phosphate dehydrogenase	Participates in the pentose phosphate pathway	Bhardwaj (2013)

#### Ecdysteroid hormones

**FIGURE 8:**
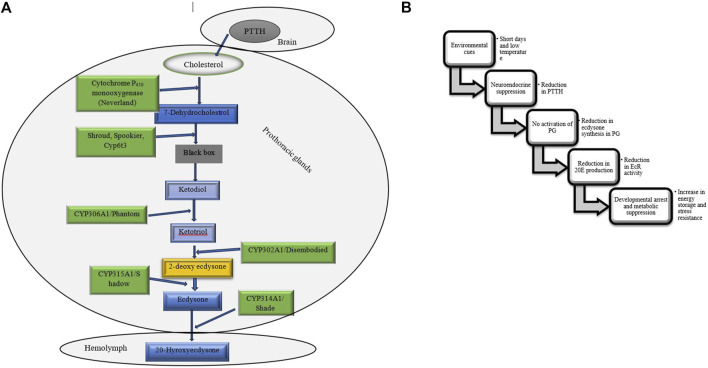
**(A)** The ecdysteroidogenic pathway synthesizes ecdysteroids, crucial hormones for molting and metamorphosis in insects. This pathway converts dietary sterols, such as cholesterol, into active hormones like 20-hydroxyecdysone through enzymatic steps. Three enzymes have been identified within the black box, although the intermediates associated with these enzymes remain undetermined (Hakeda-Suzuki and Suzuki, 2014; Niwa and Niwa, 2014). **(B)** Flowchart outlining the ecdysteroid pathway during insect diapause. PTTH, Prothoracicotropic hormone, PG, Prothoracic gland, 20E, 20-hydroxyecdysone, EcR, Ecdysone receptor (Chippendale, 1977; Denlinger et al., 2012; Chen H. et al., 2021; Karp, 2021).

**FIGURE 9:**
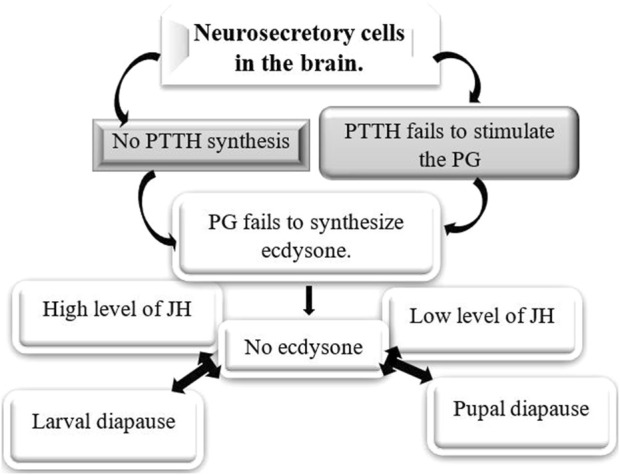
Hormonal control of larval and pupal diapause. Ecdysone plays a crucial role in the diapause of insects that enter this state as larvae or pupae, typically because of either the brain not releasing prothoracicotropic hormone (PTTH) or the prothoracic gland’s inability to respond to PTTH (Chippendale, 1977; Karp, 2021; Hutfilz, 2022).

Ecdysteroids, particularly 20-hydroxyecdysone (20E), are the principal hormones regulating molting, metamorphosis, and diapause in insects. During larval and pupal diapause, ecdysteroid levels are tightly regulated to maintain developmental arrest. The biosynthesis and metabolism of ecdysteroids are tightly regulated by different enzymes (Table 4), such as those encoded by the Halloween genes. These genes code for cytochrome P_450_ enzymes in the ecdysteroidogenic pathway, which is responsible for the biosynthesis of ecdysone from cholesterol (Gilbert et al., 2002; Gu et al., 2021). During the larval stages, ecdysone, which is the precursor of 20E, is produced in the prothoracic glands (PGs) (Figures 8A,B). In adult stages, it is mainly produced in the ovaries of females. 20E binds to the intracellular ecdysone receptor (EcR), which then leads to the formation of a receptor heterodimer. This heterodimer triggers a signaling cascade that regulates processes such as molting, metamorphosis, and reproduction (Lafont et al., 2012; Guo et al., 2021). Ecdysteroids are typically linked to the diapause of larvae and pupae. During this period, elevated JH in the hemolymph inhibits the activation of the brain-prothoracic gland axis, thereby preventing the release of ecdysteroids necessary for larval growth and pupation. In the absence of ecdysteroids, the larva is unable to initiate the next molt (Watson et al., 1987; Lonard et al., 1996; Gu et al., 1997). The failure of the PG to release ecdysteroids may result from either the brain’s inability to secrete PTTH or the prothoracic glands’ insensitivity to PTTH (Figure 9) (Mizoguchi et al., 2015; Nardiello et al., 2019). PTTH is a key neuropeptide that activates enzymes necessary for insect ecdysone biosynthesis (Figure 8A) (Denlinger, 2002; Denlinger et al., 2012; Nardiello et al., 2019). Several insect species are known to suppress PTTH and ecdysteroids during diapause. The well-known examples are: *B. mori* (Hua et al., 1999) *Chilo suppressalis* (Zhu et al., 2016), *Manduca sexta* (Watson et al., 1996), and *Heliothis virescens* (Scieuzo et al., 2018). *Pieris napi* butterflies demonstrate coordinated suppression of PTTH and ecdysteroids during diapause, reinforcing their critical role in developmental arrest (Süess et al., 2022). The reduced expression of genes responsible for ecdysteroidogenic enzymes inhibits the production of ecdysteroids, which helps to maintain developmental arrest during diapause. For example, in the cabbage armyworm, *Mamestra brassicae*, pupae that are destined for diapause exhibit a significant decrease in the expression of these enzymes. This reduction leads to a suppression of 20-hydroxyecdysone biosynthesis. However, this suppression is reversed when diapause ends, allowing development to resume (Ogihara et al., 2017). Temporal changes in gene expression related to ecdysteroid biosynthesis and downstream signaling may be different between diapause-detained and non-diapause-distained specimens. In non-diapausing eggs of *B. mori*, 20E is synthesized both from maternal conjugated ecdysteroids and via *de novo* biosynthesis. In contrast, diapausing eggs do not undergo these metabolic processes, reflecting a distinct regulatory mechanism during diapause (Gu et al., 2021). Additionally, ecdysteroid-phosphate phosphatase (EPPase) (Sonobe and Yamada, 2004), and ecdysone 20-monooxygenase (Zhou et al., 2022) influence the induction and termination of diapause. However, it is important to understand how ecdysteroids, diapause, and their regulatory enzymes interact to comprehend insect development, physiology, and their ability to adapt to changing environments. An increase in the activity of these enzymes signals the end of diapause and the resumption of growth (Niwa and Niwa, 2014). The two proteins, ultraspiracle protein (USP) and ecdysone receptor (EcR) form a complex with ecdysone. This complex binds directly to ecdysone response elements, leading to specific gene expression. For example, in the flesh fly, *Sarcophaga crassipalpis*, upregulation of USP leads to the initiation of adult development that marks the end of pupal diapause. Therefore, the expression of both proteins is crucial for receiving the ecdysteroid signal and analyzing their expression patterns is essential for understanding their role in regulating diapause ((Rinehart et al., 2001). Moreover, results highlighted the significant roles of ecdysone-signaling during the early embryogenesis of *Blattella germanica* (Cruz et al., 2023). In the cricket species *Allonemobius socius*, proteins involved in ecdysteroid synthesis and signaling, such as CYP_450_, AKR, and RACK1, are consistently upregulated during the pre-diapause phase, followed by significant downregulation later during diapause. This pattern suggests that elevated levels of ecdysone may facilitate the onset of diapause (Reynolds and Hand, 2009).

**TABLE 4:** Enzymes play a role in the metabolism of ecdysteroid hormones during insect diapause.

Regulatory enzyme	Function	References
Cytochrome P_450_ monooxygenases	Perform oxidation and reduction reactions	Danielson (2002)
Ecdysteroid biosynthetic enzymes	Regulating ecdysone biosynthesis in prothoracic glands	Niwa and Niwa (2014), Kamiyama and Niwa (2022)
Ecdysone 20-hydroxylase (Ecdysone 20-monooxygenase)	Catalyzes the chemical reaction that converts ecdysone into 20-hydroxyecdysone	Rauschenbach et al. (2008)
Ecdysteroid-phosphate phosphatase	Converts phosphate esters into active ecdysteroids	Yamada and Sonobe (2003)

**FIGURE 10:**
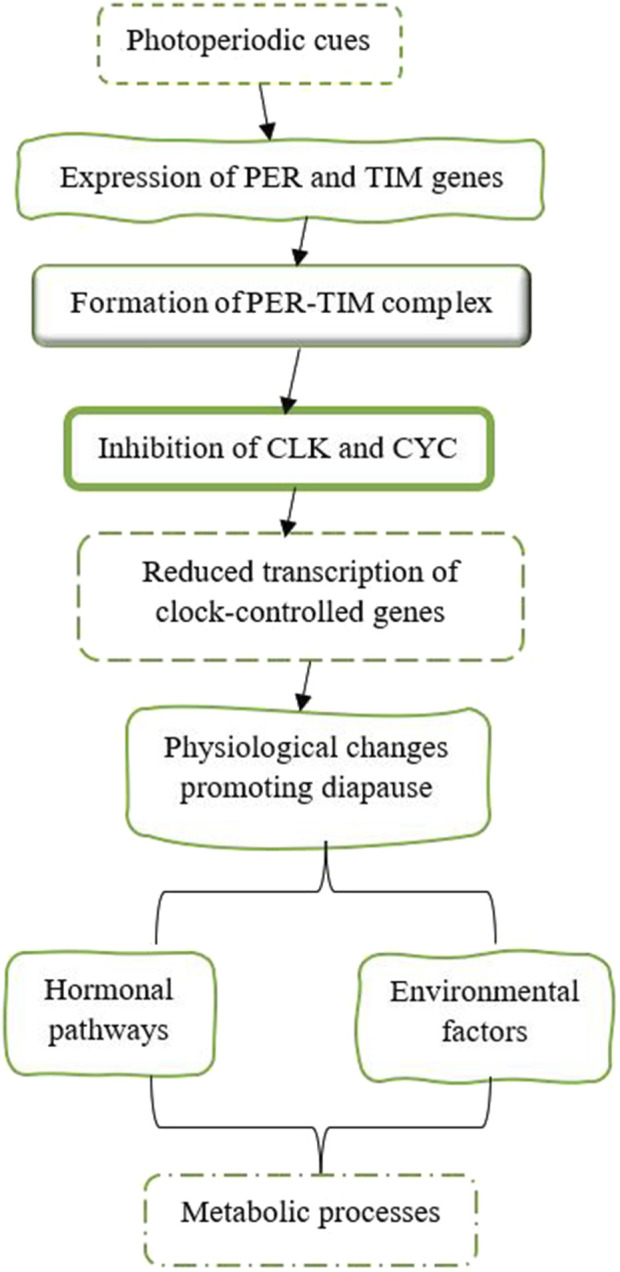
The expression of the PER and TIM genes is affected by photoperiod cues, which are crucial for starting diapause in various insects (Beck, 2007; Mcleod and Beck, 2007; Shimokawa et al., 2008; Ikeno et al., 2010; Vesala and Hoikkala, 2011; Vesala et al., 2012; Saunders, 2014; Anduaga et al., 2018; Goto and Nagata, 2022).

The circadian clock, which controls daily rhythms in physiology and behavior, plays a crucial role in the regulation of diapause. Two core components of the circadian clock in insects are Period (PER) and Timeless (TIM) (Figure 10). These proteins operate within a negative feedback loop that modulates the expression of other clock genes, such as Clock (CLK) and Cycle (CYC) (Fyie et al., 2024; Goto and Nagata, 2022; Ikeno et al., 2010). The interaction among these genes establishes rhythmic patterns of gene expression that influence various physiological processes. PER accumulates during the night, and when it reaches a certain threshold, it moves into the nucleus, where it dimerizes with TIM. Together, they inhibit the activity of CLK and CYC, which reduces the transcription of their associated genes as well as other clock-controlled genes. Similar to PER, TIM also accumulates in response to light-dark cycles and plays an important role in stabilizing the PER protein. The formation of the PER-TIM complex is essential for maintaining circadian rhythms and is involved in photoperiodic signaling that can trigger diapause (Fyie et al., 2024; Goto and Nagata, 2022; Ikeno et al., 2010). The expression of the PER and TIM genes is influenced by photoperiodic cues, which are essential for initiating diapause in many insects. Shorter day lengths, indicative of autumn, trigger an increased expression of these clock genes, leading to physiological changes that promote diapause. For example, studies have demonstrated that silencing the Clock gene disrupts normal diapause responses in crickets, highlighting its role in sensing photoperiod and regulating reproductive behaviors associated with diapause (Goto and Nagata, 2022). In larvae of the sugar beet moth, *Scrobipalpa ocellatella*, destined for diapause, short-day conditions resulted in increased levels of the PER and TIM genes. These elevated gene levels decreased the amount of PTTH in both the brain and hemolymph. This reduction in PTTH then led to lower levels of 20E and triggered the induction of diapause (Ahmadi et al., 2021). Research has shown that the genes PER and TIM are involved in the temperature-dependent induction of diapause. In species such as *B. mori*, mutations in these clock genes can impair their ability to enter diapause when exposed to different temperature conditions. This indicates that the circadian clock combines both photoperiodic and temperature signals to regulate diapause (Homma et al., 2022). The activity of the proteins PER and TIM is closely associated with hormonal pathways that regulate metabolism during diapause. By influencing hormonal pathways and metabolic processes, PER and TIM help determine when an insect enters or exits diapause, thereby ensuring its survival during adverse conditions. For instance, fluctuations in JH levels can interact with circadian clock mechanisms, influencing an insect’s decision to enter or exit diapause (Ikeno et al., 2010). Understanding this relationship offers valuable insights into how insects adapt their life cycles in response to changing environments. Maintaining circadian rhythms during diapause is essential for properly timing metabolic processes. Disruptions to these rhythms, caused by environmental factors like artificial light at night (ALAN), can interfere with the onset of diapause by altering the expression patterns of clock genes such as PER and TIM (Fyie et al., 2024).

#### Prothoracicotropic hormone (PTTH)

Proper regulation of prothoracicotropic hormone (PTTH) is crucial for determining whether an insect resumes development or enters diapause. PTTH functions as a developmental trigger after diapause by being released into the hemolymph before the activation of the prothoracic glands, which then synthesize the necessary ecdysteroids for metamorphosis. In other words, timely PTTH release signals the end of diapause and initiates the subsequent developmental processes (Denlinger, 2002; Denlinger et al., 2012; Yamada et al., 2016). For example, the larvae of the cabbage army moth, *M. brassicae*, destined for diapause have lower levels of PTTH in their blood and reduced PTTH gene expression several days before they pupate. This suggests that the diapause program is already established in PTTH neurons during the middle to final stage of the larvae’s development (Mizoguchi et al., 2013). Co-regulation of diapause by PTTH and ecdysone has also been reported in *Helicoverpa armigera* (Hou and Xu, 2007) and *P. napi* (Süess et al., 2022). However, no information about the enzyme activity and its impacts on PTTH metabolism during diapause is available.

#### Insulin-like peptides (ILPs)

Insulin-like peptides (ILPs) are multifunctional hormones that play a crucial role in regulating metabolic pathways in insects, particularly in glycolysis, through complex signaling networks (Fernandez and Torres-Alemán, 2012). This interaction is essential for various physiological processes, including growth, reproduction, and energy balance. ILPs are encoded by multigene families and are expressed in several tissues, such as the brain, midgut, salivary glands, and fat body. Their expression is often specific to certain tissues and developmental stages, allowing for precise control of metabolism throughout different life stages of insects (Brown et al., 2008; Kawabe et al., 2019; Chowański et al., 2021). When secreted, ILPs act as hormones that bind to insulin receptors (IRs) and activate intracellular signaling pathways. ILPs activate phosphoinositide 3-kinase (PI3K) and protein kinase B (Akt), which leads to the phosphorylation and inhibition of glycogen synthase kinase-3 (GSK-3). This process enhances glycogen breakdown and increases glucose availability for glycolysis. Additionally, ILPs promote the expression of key glycolytic enzymes, such as hexokinase, phosphofructokinase, and pyruvate kinase, thereby accelerating glycolysis and ATP production (Chowański et al., 2021; Weger and Rittschof, 2024). In *D. melanogaster*, insulin-like peptides (ILPs) increase the expression of Glut1-like glucose transporters, which facilitates glucose uptake into cells and supports glycolysis (Kauffman and DiAngelo, 2024). Reduced ILPs signaling is associated with diapause induction, as it shifts metabolism from anabolic to energy-conserving pathways. During periods of high ILPs signaling (the fed state), insects prioritize glycolysis for rapid energy production. Conversely, during diapause or starvation (low ILPs signaling), metabolism shifts toward lipid oxidation and gluconeogenesis to conserve carbohydrate reserves (Wu and Brown, 2006; Chowański et al., 2021; Weger and Rittschof, 2024). In the mosquito, *Culex pipiens* and the fruit fly, *D. melanogaster*, the levels of two specific insulin-like peptides, ILP-1 and ILP-5, decrease during diapause. This highlights their role in promoting lipid storage and enhancing stress resistance during this adaptive physiological state (Sim and Denlinger, 2009). The insulin/insulin-like growth factor signaling (IIS) pathway is essential for regulating carbohydrate and lipid metabolism, and plays a pivotal role in the regulation of diapause (Sim and Denlinger, 2013). In *C. pipiens* IIS signaling opposes diapause (Sim and Denlinger, 2013). Glucagon- and insulin-like signaling are vital in the physiological changes observed in *D. melanogaster* during reproductive diapause (Kubrak et al., 2014). Trehalose accumulation is also linked to the IIS pathway (Li et al., 2021). For instance, in *Drosophila*, ILPs also regulate trehalose metabolism, ensuring a steady supply of glucose for glycolysis. The enzyme trehalase, which converts trehalose into glucose, is regulated by the IIS pathway that links nutrient levels to essentially biological processes. In the Chinese oak silkworm, *Antheraea pernyi*, bovine insulin can initiate the end of diapause by increasing trehalose breakdown during the diapause termination phase. This indicates a regulatory mechanism that connects hormonal signals with trehalose metabolism during diapause (Li et al., 2021). The expression of insulin pathway genes in the solitary bee, *Megachile rotundata*, is significantly influenced by overwintering conditions during diapause. Bees that overwinter under fluctuating temperatures show different insulin signaling profiles compared to those kept at constant temperatures, suggesting that environmental conditions modulate insulin signaling during diapause (Cambron et al., 2021). In *B. mori*, ILPs influence glycolysis during metamorphosis, optimizing carbohydrate utilization to meet developmental energy demands (Chowański et al., 2021). Insulin-like growth factor signaling is a crucial regulatory pathway in insect diapause. It influences energy metabolism, stress resistance, and developmental decisions allowing insects to survive harsh environmental conditions.

#### Allatostatins and allatotropins

Allatostatins (ASTs) are a family of neuropeptides that primarily inhibit the synthesis of juvenile hormone (JH). By inhibiting JH synthesis, allatostatins help regulate developmental processes during diapause. Allatotropins are another class of neuropeptides that stimulate the production of JH (Sheng et al., 2007). Insect diapause is tightly regulated by JH levels, which are influenced by allatotropin and allatostatin neuropeptides. In *C. pipiens*, suppression of allatotropin is critical for inducing reproductive diapause. Experimental knockdown of allatotropin mimics diapause-like states by halting ovarian development, reversible with JH application (Kang et al., 2014). The balance between allatotropin and allatostatin activity is central to regulating diapause through their control of JH levels, affecting both metabolic suppression and developmental arrest. In addition to the previously mentioned hormones, other hormones such as hypertrehalosemic hormones (HTHs) and adipokinetic hormones (AKHs) play a role in mobilizing lipids and carbohydrates from the insect’s fat bodies during intense physical activities. These hormones, along with their regulatory enzymes, may also be involved in insect diapause; however, there is currently no available information on this topic.

#### Carbohydrates, diapause, and regulatory enzymes

Diapause is characterized by metabolic flexibility in response to environmental temperature changes (Uzelac et al., 2020). Carbohydrates are essential for energy storage and metabolic adjustments for maintaining and terminating diapause. Additionally, they serve as cryoprotectants, helping to survive during diapause. Regulating enzyme activity during diapause is essential for energy balance (Table 5). Research shows that glycolysis and gluconeogenesis enzymes (Figure 3B) are closely controlled through allosteric modifications and gene expression changes, allowing the organism to adjust energy use and storage during dormancy (Weber et al., 1967; Kageyama and Ohnishi, 1971; Ishii et al., 2004). Trehalose and glycogen are the key sugars of insects, playing crucial roles in energy metabolism and ecological protection (Guo et al., 2015). The conversion between glycogen and trehalose has been demonstrated in various diapausing species, such as the pistachio seed wasp, *Eurytoma plotnikovi* (Mohammadzadeh et al., 2017), the pistachio white leaf borer, *Ocneria terebinthina* (Behroozi et al., 2012), the almond wasp, *Eurytoma amygdali* (Khanmohamadi et al., 2016), the Sunn pest, *Eurygaster integriceps* (Hasanvand et al., 2020), the silkworm, *Philosamia cynthia pryeri* (Hayakawa and Chino, 1981), and four *Drosophila* species (Kimura et al., 1992). This conversion is often accompanied by changes in enzyme activities. Trehalose synthases are crucial enzymes for converting trehalose into glycogen (Figure 3A). Among them, trehalose-6-phosphate synthase is a vital enzyme involved in trehalose synthesis (Pan et al., 2008; Zhang et al., 2023). For example, in *Lissorhoptrus oryzophilus*, trehalose-6-phosphate synthase improves cold tolerance by facilitating rapid cold hardening through trehalose accumulation (Zhang et al., 2023). The expression of trehalose-6-phosphate synthase, glycogen synthase, and glycogen phosphorylase in *Aphidius gifuensis* was inhibited during diapause maintenance. The suppression of trehalose-6-phosphate synthase expression indicates a decreased synthesis of trehalose, which may help avoid unnecessary energy expenditure. Additionally, the downregulation of glycogen synthase implies a shift away from active glycogen storage processes, which is consistent with the metabolic stasis characteristic of diapause. By reducing the expression of glycogen phosphorylase, glycogen degradation is minimized, indicating that glycogen may play a significant role in development after diapause (Zhang et al., 2020). Increased trehalase activity during diapause termination helps lower trehalose levels, facilitating the transition out of diapause. In simpler terms, reduced trehalose levels are necessary to end diapause (Guo et al., 2015). Glycogen can be rapidly converted into dextrose or trehalose for energy and transported to other tissues for glycolytic fuel (Arrese and Soulages, 2010). Glycogenesis and glycogenolysis are regulated by two key enzymes glycogen synthase and glycogen phosphorylase, respectively (Figures 3A,B). Enzymes like phosphofructokinase, phosphoglucomutase, and pyruvate kinase regulate glycolysis, controlling both glycogen and trehalose metabolism in insects, while glucose-6-pyrophosphate dehydrogenase, phosphoglucomutase, and phosphoenolpyruvate carboxykinase play a role in gluconeogenesis, ensuring a balance of energy production and utilization (Pan et al., 2020). Phosphoenolpyruvate carboxykinase (PEPCK) is an enzyme belonging to the lyase family, playing a crucial role in the metabolic pathway of gluconeogenesis (Yang J. et al., 2009). It catalyzes the conversion of oxaloacetate into phosphoenolpyruvate and carbon dioxide. The rate-limiting step of gluconeogenesis is PEPCK, which is generally recognized as the first committed step in this pathway (Martins da Silva et al., 2023). Research has shown that PEPCK activity can be upregulated in diapausing insects, facilitating gluconeogenesis and enabling the mobilization of stored lipids and carbohydrates as needed. For example, in the flesh fly, *Sarcophaga bullata*, two isoforms of PEPCK show different expression patterns throughout development and in response to environmental stresses such as cold and starvation. This indicates that specific isoforms may be preferentially activated during diapause to meet metabolic needs (Roncalli et al., 2018; Martins da Silva et al., 2023) In pupae of the cotton bollworm, *H. armigera*, destined for diapause, low pyruvate levels in the brains are linked to three enzymes: pyruvate kinase (PK), phosphoenolpyruvate carboxykinase (PEPCK), and phosphoglycerate mutase (PGAM) (Wang et al., 2018). In addition to the previously mentioned enzymes, the activities of leucine aminopeptidase and alkaline phosphatase, both of which are associated with glucose metabolism, decrease during diapause. Furthermore, lactate dehydrogenase and glyceraldehyde-3-phosphate dehydrogenase exhibit complete activity loss by the late diapause stage. These findings suggest that the glycolytic pathway is disrupted during diapause (Sluss et al., 1975).

**TABLE 5:** Enzymes play a role in the metabolism of carbohydrates during insect diapause.

Regulatory enzyme	Function	References
Trehalose-6-phosphate synthase	Catalyzes the synthesis of trehalose 6-phosphate	Fichtner et al. (2020), Chen X. et al., 2021
Glycogen synthase	The key enzyme in the conversion of glucose into glycogen (glycogenesis)	Buschiazzo et al. (2004)
Glycogen phosphorylase	Catalyzes the rate-limiting step in glycogenolysis by releasing glucose-1-phosphate from the terminal alpha-1,4-glycosidic bond	BhagavanN (2002)
Trehalose-6-phosphate phosphatase	Hydrolyses the trehalose 6-phosphate into trehalose and phosphate	Kerbler et al. (2023)
Trehalase	A glycoside hydrolase catalyzes the conversion of trehalose to glucose	Terra et al. (2019)
Phosphofructokinase	In glycolysis, phosphofructokinase catalyzes the phosphorylation of fructose-6-phosphate (F6P) using ATP as a phosphate donor to form fructose-1,6-diphosphate and ADP.	Gheshlaghi et al. (2009)
Phosphoglucomutase	In glycolysis, catalyzes the interconversion of glucose 1-phosphate (G-1-P) and glucose 6-phosphate (G-6-P)	Brolin and Berne (1970)
Pyruvate kinase	In glycolysis, converts glucose into ATP	van Dijk et al. (2023)
Glucose-6-phosphate dehydrogenase	Participates in the pentose phosphate pathway by catalyzing D-glucose 6-phosphate into 6-phospho-D-gluconic-1,5-lactone	Thomas et al. (1991)
Phosphoenolpyruvate carboxykinase	In gluconeogenesis, catalyzes the conversion of oxaloacetate into phosphoenolpyruvate	Méndez-Lucas et al. (2014)
Aldose reductase	Initiates the NADPH-dependent conversion of glucose to sorbitol in the polyol pathway of glucose metabolism	Attar et al. (2013)
Polyol dehydrogenase	Catalyzes a two-step process converting glucose to fructose. Glucose is first reduced to sorbitol and then oxidized to fructose	Bonnefont-Rousselot (2002)
Ketose reductase	A NAD-dependent polyol dehydrogenase catalyzes the NADH-dependent reduction of fructose to sorbitol	Doehlert (1987)
Fructose-1,6-bisphosphatase	a key enzyme in glycolysis that catalyzes the hydrolysis of D-fructose 1,6-bisphosphate to D-fructose 6-phosphate and inorganic phosphate	Cui and Tcherkez (2021)
Glyceraldehyde-3-phosphatase	a crucial glycolytic enzyme that facilitates the reversible conversion of glyceraldehyde-3-phosphate into 1,3-diphosphoglycerate	Perez-Casal and Potter (2016)

#### Metabolism, diapause, and regulatory enzymes

A hallmark of diapause is the dramatic reduction in metabolism, orchestrated by an array of molecular mechanisms that mirror those observed in proliferating cells. This metabolic slowdown is driven by comprehensive regulatory processes, including transcriptional, post-transcriptional, and post-translational modifications, that modulate gene expression, mRNA and protein accumulation, and protein function. In tandem, shifts in biochemical pathways and metabolic products enhance the organism’s stress tolerance, ensuring survival during adverse conditions (Hahn and Denlinger, 2007; MacRae, 2010; Huang et al., 2022). Energy expenditure during diapause varies across insect species and is influenced by factors such as diapause duration, body size, and environmental conditions. Energy expenditure during diapause initially shows low metabolic activity as energy is conserved. Over time, metabolic rates gradually increase, reflecting the utilization of stored energy to maintain essential metabolic functions (Hahn and Denlinger, 2007; Hahn and Denlinger, 2010). For example, studies on various beetle species have shown that their metabolic intensity increases as they advance through diapause, leading to more rapid use of energy reserves (Lehmann et al., 2020a). The study examined the relationship between metabolic rate and temperature in dormant willow leaf beetles, *Chrysomela aeneicollis,* and found that the metabolic rate increased as diapause progressed. Additionally, acclimating the beetles to variable conditions affected both the intensity of their metabolism and their sensitivity to temperature changes (Roberts and Williams, 2022). Enzymes involved in fatty acid synthesis and triacylglycerol storage play a pivotal role in the initiation phase of diapause. Once diapause is established, metabolic pathways such as fatty acid biosynthesis, oxidative phosphorylation, and carbohydrate metabolism, along with their regulatory enzymes, are often downregulated to conserve energy (Zhang et al., 2019). β-Oxidation is the catabolic process that breaks down fatty acids to produce acetyl-CoA, NADH, and FADH_2_. The key enzymes in β-oxidation are acyl-CoA dehydrogenase, enoyl-CoA hydratase, hydroxyacyl-CoA dehydrogenase, and ketoacyl-CoA thiolase (Tables 6, 7). The activity of these enzymes can be affected by hormonal changes and the insect’s physiological state, ensuring energy mobilization meets the insect’s metabolic needs during dormancy (Wanders et al., 1999). For instance, in *C. pipiens*, fatty acid synthase is upregulated during early diapause, whereas genes involved in β-oxidation and energy generation are downregulated (Sim and Denlinger, 2009). Moreover, enzymes involved in fatty acid synthesis are suppressed during the overwintering of the mosquito, *A. albopictus* (Poelchau et al., 2013). Insects that undergo diapause may show reduced oxidative metabolism, characterized by decreased citrate synthase activity and lower levels of total glutathione and lipid peroxidation (Moreira et al., 2012). Additionally, to enhance metabolic activity before diapause, telomerase activity might be upregulated (Koubová et al., 2019). The relationship between diapause and cold tolerance is essential for regulating metabolic enzyme activity in various insect species. For instance, in the European corn borer, *O. nubilalis*, enzyme activity aligns with temperature changes, demonstrating these insects’ adaptive responses to their environment. Research also shows that during diapause, specific enzymes such as glucose-6-phosphate dehydrogenase, lactate dehydrogenase, and aldose reductase become more active, which aids in the synthesis of protective metabolites, whereas, in some species diapause is marked by reduced activity of energy-intensive enzymes like ATPases and phosphofructokinase, reflecting a general metabolic slowdown (Uzelac et al., 2020; Moreira et al., 2021). Moreover, the activity of certain other enzymes may vary during diapause. For instance, in diapause-destined pupae of *H*. *armigera*, low glycogen synthase kinase 3β (GSK3β) activity is caused by elevated serine/threonine protein kinase activity, leading to diapause initiation (Song et al., 2023). In diapausing larvae of the sunflower caterpillar, *Homoeosoma ellectellum*, the oxidative metabolism and peroxide-detoxifying enzymes are downregulated, while glutathione transferase and isocitric dehydrogenase are upregulated (Moreira et al., 2021). The study revealed that cytochrome oxidase serves as the main terminal oxidase during the diapause of *Cecropia*, *Cynthia, Promethea*, and *Polyphemus* silkworm pupae (Kurland and Schneiderman, 1959).

**TABLE 6:** Enzymes play a role in the metabolism of lipids during insect diapause.

Regulatory enzyme	Function	References
Acyl-CoA dehydrogenase	Creates a double bond between the second and third carbons down from the CoA group on acyl-CoA and in the process produces an FADH2	Swigoňová et al. (2009)
Enoyl-CoA hydratase	Hydrates the double bond between the second and third carbons on 2-trans/cis-enoyl-CoA	Bennett et al. (2020)
Hydroxy acyl-CoA dehydrogenase	catalyzes the NAD + dependent dehydrogenation of 3-hydroxy acyl–CoA to 3-ketoacyl-CoA in the β-oxidation of fatty acids	Galcheva et al. (2018)
Ketoacyl-CoA thiolase	Catalyzes the reversible hemolytic cleavage of 3-ketoacyl-CoA into acyl-CoA and acetyl-CoA	Fox et al. (2014)
Fatty acid synthase	Multi-enzyme polypeptides that catalyze fatty acid synthesis (the synthesis of palmitate from acetyl-CoA and malonyl-CoA, in the presence of NADPH.	Fox et al. (2014)
Citrate synthase	This enzyme catalyzes the formation of citrate from acetyl CoA and oxaloacetate in the Krebs cycle	Huang et al. (2020)
Telomerase	The synthesis of a telomere involves a reverse transcriptase called telomerase, which acts as an RNA-dependent DNA polymerase	Shay and Wright (2019)
Lactate dehydrogenase	Catalyzes the reversible conversion of lactate into pyruvate	Kantor et al. (2001)
Alanine aminotransferase	Catalyzes the transfer of an amino group from L-alanine to α-ketoglutarate, resulting in a reversible transamination reaction that produces pyruvate and L-glutamate	Yang et al. (2009b)
Aspartate aminotransferase	This enzyme catalyzes the conversion of aspartate and α-ketoglutarate into oxaloacetate and glutamate	Kirsch et al. (1984)
Cytochrome c oxidase	catalyzes the four-electron reduction of oxygen molecules to water and utilizes the chemical energy to transport four protons across cell membranes	Ishigami et al. (2023)
Glycogen synthase kinase 3β	A kinase that phosphorylates several substrates and plays a crucial role in various cellular signaling pathways	Beurel et al. (2015)
ATPases	A group of enzymes catalyzes the synthesis of ATP from ADP.	Hisabori (2020)
Peroxide-detoxifying enzymes	Catalyzing the conversion of hydrogen peroxide to water (peroxiredoxins, glutathione peroxidases, and catalase)	Stancill and Corbett (2023)
Glutathione transferase	This enzyme catalyzes the conjugation of reduced glutathione with substrates	Lin and Gerson (2014)
Isocitrate dehydrogenase	Catalyzes the oxidative decarboxylation of isocitrate to α-ketoglutarate in Kreb’s cycle, during which NAD+ is reduced to NADH or NADP + to NADPH	Liu et al. (2024)

**TABLE 7:** The four enzymes are crucial for the β-oxidation process, breaking fatty acids into acetyl-CoA molecules for energy production.

Enzyme	Function	Activity during diapause	Key products	References
Acyl-CoA dehydrogenase	Catalyzes the oxidation of acyl-CoA to trans-2-enoyl-CoA, producing FADH2	Exhibit altered expression levels during diapause preparation and maintenance. Upregulation during unfavorable conditions may enhance the insect’s ability to utilize stored fats efficiently	Trans-2-enoyl-CoA, FADH2	Liu et al. (2016), Tan et al. (2017), Zhang et al. (2019), Lehmann et al. (2020b), Xiang et al. (2021), Yang et al. (2023)
Enoyl-CoA hydratase	Adds a water molecule to trans-2-enoyl-CoA, forming L-3-hydroxyacyl-CoA	The physiological requirements influence the expression levels during diapause. The activity at the end of diapause enhances the effective transformation of fatty acids into energy	L-3-hydroxyacyl-CoA	
Hydroxyacyl-CoA dehydrogenase	Oxidizes L-3-hydroxyacyl-CoA to 3-ketoacyl-CoA, generating NADH in the process	Differentially expressed genes involved in fatty acid metabolism are essential for lipid accumulation during the preparation phase for diapause	3-ketoacyl-CoA, NADH	
Ketoacyl-CoA thiolase	Cleaves 3-ketoacyl-CoA to yield acetyl-CoA and acyl-CoA, allowing the cycle to continue	Differentially expressed in response to the physiological requirements of diapause	Acetyl-CoA, acyl-CoA	

#### Metabolic flexibility

**FIGURE 11:**
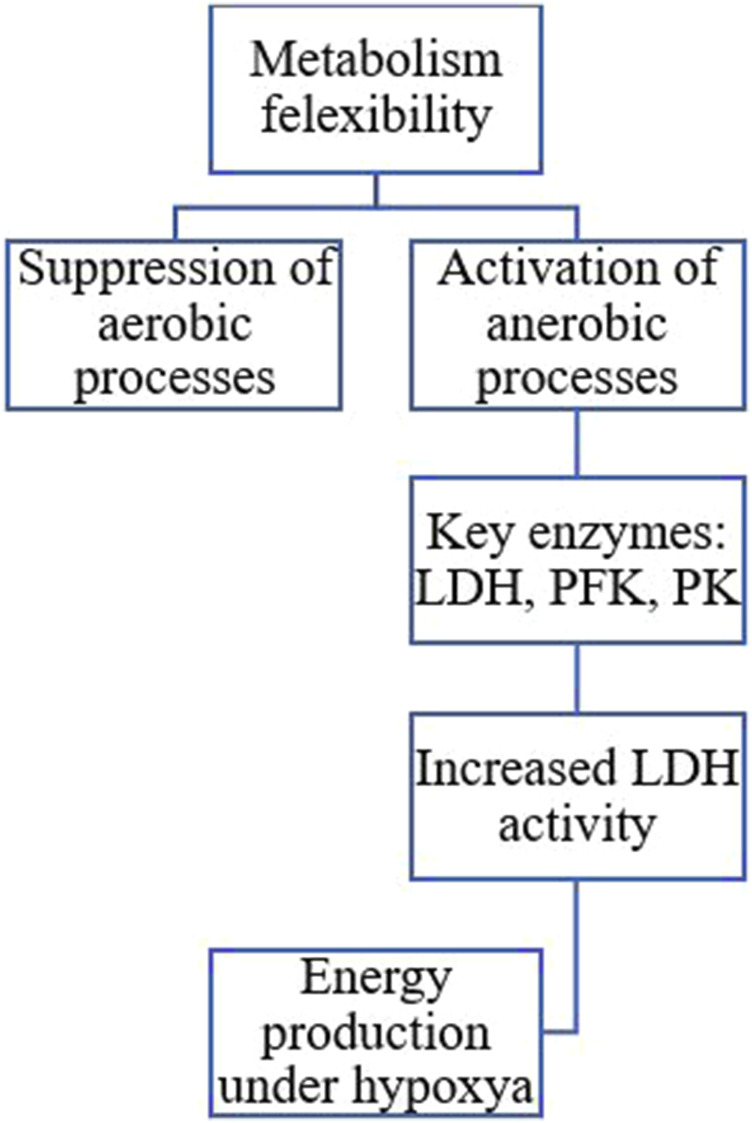
The transition to anaerobic metabolism is marked by increased enzymes that enhance glycolysis and lactate production. Key enzymes involved in this process include lactate dehydrogenase (LDH), phosphofructokinase (PFK), and pyruvate kinase (PK) (Hahn and Denlinger, 2007; Hahn and Denlinger, 2010; Sinclair, 2015; Uzelac et al., 2020; Chen X. et al., 2021).

Insects display remarkable metabolic flexibility during diapause, allowing them to manage their energy reserves effectively (Figure 11). They can sense their energy levels and adjust their metabolic pathways accordingly (Hahn and Denlinger, 2010; Lehmann et al., 2016). For instance, aerobic processes are suppressed, as indicated by the reduced activity of enzymes associated with the Krebs cycle, while anaerobic pathways become more prominent. In the wheat midge, *Sitodiplosis mosellana* (Diptera: Cecidomyiidae), reduced activity of the citric acid cycle and increased production of functional metabolites likely contribute to maintaining low metabolic activity and cold tolerance during diapause (Huang et al., 2022). Research shows that the transition to anaerobic metabolism is marked by an increase in enzymes that enhance glycolysis and lactate production. Key enzymes involved in this process include lactate dehydrogenase (LDH), phosphofructokinase (PFK), and pyruvate kinase (PK). These enzymatic adaptations are essential for maintaining energy production during hypoxia or metabolic stress (Rodas et al., 2000). Studies on the diapausing larvae of the European corn borer, *O. nubilalis*, have revealed elevated lactate dehydrogenase activity levels at near-freezing temperatures. This indicates a shift towards anaerobic metabolism, enabling insects to produce energy without relying on oxygen, especially when oxygen availability is limited due to cold conditions (Uzelac et al., 2020). Diapausing pupae of the flesh fly, *Sarcophaga* sp. utilizes both aerobic lipid catabolism and anaerobic glycolysis to meet their energy demands (Chen C. et al., 2021). The metabolic switch from aerobic to anaerobic processes during insect diapause is a complex adaptation that helps these organisms survive harsh environments while efficiently managing their energy reserves (Lehmann et al., 2016; Uzelac et al., 2020).

#### Cryoprotectants, diapause, and regulatory enzymes

Cryoprotectants protect living cells and tissues from damage caused by freezing temperatures (Elliott et al., 2017; Jaiswal and Vagga, 2022; Chen et al., 2023). Insects have developed different mechanisms to survive in cold environments, including producing and accumulating cryoprotectants. The capacity of insects to accumulate these cryoprotectants changes during diapause development and involves various regulatory enzymes (Storey and Storey, 1991; Hahn and Denlinger, 2007). It was previously thought that insects accumulate cryoprotectants mainly from their internal macromolecular reserves during cold acclimation. However, the research indicates that food intake is essential for providing the materials necessary to create cryoprotectants, challenging the notion that these compounds mainly originate from the insect’s internal reserves (Moos et al., 2022). Moreover, cold-acclimated *Chymomyza costata* (Diptera: Drosophilidae) larvae use amino acids from their food to produce proline, a key cryoprotectant and also convert internal reserves of glutamine. Various enzymes (Table 5) play a crucial role in regulating the metabolic pathways that lead to the accumulation of insect cryoprotectants. The interaction between glycogen breakdown, sugar metabolism, and amino acid conversion is essential for producing effective cryoprotectants like trehalose and glycerol (Wang et al., 2016). Trehalose-6-phosphate synthase, glycogen phosphorylase, phosphofructokinase, aldose reductase, polyol dehydrogenase, ketose reductase, and glucose-6-phosphate dehydrogenase are among the most important of them (Yocum et al., 2009; Kim et al., 2017; Smolinski et al., 2019; Song et al., 2023). The activity of these enzymes may increase in the early phase of diapause but stabilize or decrease in the late phase. However, cold treatment affects enzyme activity differently based on the diapause phase, with some enzymes showing increased activity and others decreasing (Koštál et al., 2004). Cold acclimation involves significant physiological changes, including increased trehalose-6-phosphate synthase activity and related metabolic pathways. This increase is associated with the production of trehalose, an essential cryoprotectant that helps improve ion and water balance during exposure to cold temperatures. This balance is crucial for maintaining cellular integrity and function under stress conditions (Zhang et al., 2023). A study on the effects of cold acclimation during diapause showed that the activity of specific metabolic enzymes remains in dynamic equilibrium and is synchronized with external factors like low temperatures. This adaptation allows the organism to adjust its metabolism in response to environmental changes, directing it toward producing protective compounds such as glycerol and alanine (Uzelac et al., 2020). For instance, in *Trichogramma dendrolimi*, decreasing temperatures led to increased enzyme activity and trehalose accumulation (Guo et al., 2015). Three enzymes, aldose reductase, ketose reductase, and polyol dehydrogenase, were examined in adult female linden bugs, *Pyrrhocoris apterus*. High enzyme activity during female diapause indicates a strong ability to synthesize and store polyol cryoprotectants (Tollarova, 2008). Low temperatures cumulatively enhance glycerol production in the larvae of the rice stem borer, *C. suppressalis*, during various diapause development phases. This glycerol accumulation results from the activation of glycogen phosphorylase, inhibition of fructose-1,6-bisphosphatase, and activation of enzymes linked to glycerol synthesis, particularly glyceraldehyde-3-phosphatase (Table 8) and polyol dehydrogenase with glyceraldehyde activity (Li et al., 2002). However, cold hardiness is often different from the diapause program and may be triggered by the low temperatures of winter, rather than by the diapause program (Khanmohamadi et al., 2016).

**TABLE 8:** Antioxidant enzymes and their function in insect diapause.

Regulatory enzyme	Function	References
Superoxide dismutase	Catalyzes the dismutation of superoxide anion to hydrogen peroxide and triplet oxygen	Deeth (2021)
Catalase	Hydrogen peroxide (H2O2) is broken down into water and oxygen molecules, protecting (Singh and Kumar, 2019) cells against oxidative damage caused by reactive oxygen species	Singh and Kumar (2019)
Peroxidases	Enzymes that catalyze the oxidation of substrates using hydrogen peroxide or organic peroxides as oxidizing agents	Everse (2013)
Glutathione S-transferase	Catalyzing the conjugation of electrophilic substrates to glutathione to detoxify endogenous compounds	Sheehan et al. (2001)
Isocitrate dehydrogenase-NADP+	Catalyzing the oxidative decarboxylation of isocitrate to α-ketoglutarate, while reducing NAD(P)+ to NAD(P)H	Reitman and Yan (2010)

#### Antioxidant enzymes and diapause

During diapause, insects enter a dormant state characterized by low metabolic activity and diminished antioxidant defenses. To counteract the resulting increase in oxidative stress, antioxidant enzymes are often upregulated. This adjustment helps protect cellular components from damage. The dynamic regulation of these enzymes, through changes in their expression and activity, enables insects to adapt to the physiological challenges of dormancy (Table 8) (Moreira et al., 2021). Enzymes like superoxide dismutase, catalase, and peroxidases help scavenge harmful reactive oxygen species (ROS) and protect cells from damage during diapause (Moreira et al., 2021). Studies have shown that the activity of antioxidant enzymes can significantly differ between the diapausing and non-diapausing stages of different insect species. For instance, in the European corn borer, *O. nubilalis*, diapausing larvae exhibited reduced activities of catalase and glutathione S-transferase (GST) compared to non-diapausing larvae, suggesting a different oxidative stress management strategy during dormancy (Jovanović-Galović et al., 2007). However, antioxidant enzyme levels alone cannot fully indicate oxidative stress. Levels of glutathione and the reduced to oxidized glutathione ratio (GSH: GSSG) are also key markers. Additionally, a significant increase in the activities of GST and isocitrate dehydrogenase-NADP+ during diapause, suggests a preparation for potential oxidative stress (Moreira et al., 2021). The research on the sunflower caterpillar, *Chlosyne lacinia*, reveals significant insights into the metabolic adaptations associated with diapause. The study found that diapausing larvae exhibit a decreased capacity to manage oxidative stress, primarily due to reduced activities of peroxide-decomposing antioxidant enzymes. This impairment is critical as it suggests that these larvae are less equipped to handle oxidative damage during this dormant phase. However, the elevated levels of glutathione transferase and isocitrate dehydrogenase-NADP+ in diapausing larvae indicate a preparatory response to potential oxidative stress. The upregulation of these enzymes suggests that, despite their reduced overall antioxidant capacity, the caterpillars are strategically enhancing specific pathways to mitigate oxidative damage during diapause in tropical environments. Overall, the findings highlight the intricate balance between metabolic suppression and antioxidant defense mechanisms in diapausing insects, contributing to our understanding of their survival strategies in fluctuating habitats (Moreira et al., 2021). In the European corn borer, *O. nubilalis*, the hexose monophosphate shunt is linked to the antioxidative defense system, which is crucial for maintaining redox balance during diapause (Stanic et al., 2004). In the silkworm, *Antheraea mylitta*, diapause-destined pupae showed lower levels of hydrogen peroxide and higher activities of catalase and superoxide dismutase, indicating a strategic antioxidant protection mechanism that supports dormancy and potentially extending lifespan (Sahoo et al., 2018). Diapause adaptation to oxidative stress entails a complex interaction between antioxidant enzyme activity and ROS levels. For example, lower levels of hydrogen peroxide in diapausing insects may indicate a reduced metabolic rate and lower oxidative stress, allowing for survival during unfavorable conditions (Khurshid et al., 2021).

#### Protease enzymes and diapause

Protease enzymes (Table 9) are involved in the degradation of proteins, essential for recycling amino acids and regulating metabolic processes during diapause. Changes in the abundance of proteolytic enzymes can indicate shifts in metabolic pathways that support survival under unfavorable conditions (Richard, 2017). Studies have shown that different proteolytic enzymes and protease inhibitors exhibit varying abundance patterns in diapausing versus non-diapausing insects. For example, in the parasitoid wasp, *Nasonia vitripennis* (Hymenoptera: Pteromalidae), proteomic analyses revealed changes in the abundance of enzymes related to protein synthesis and degradation during early diapause, suggesting a metabolic switch that prepares the insect for dormancy (Wolschin and Gadau, 2009). Increased activity of proteases in post-diapause can be associated with the rebuilding of tissues and preparation for development. In the fall webworm, *Hyphantria cunea*, diapause-destined larvae exhibit lower protease activity than non-diapause-destined larvae. This reduction in protease activity helps conserve protein reserves, which is crucial for surviving the diapause period when feeding is impossible (Zhao et al., 2021). The decrease in protease activity during diapause facilitates a transition from protein breakdown to accumulating energy reserves like lipids, essential for sustaining long-term survival during dormancy. In agreement, in the mosquito, *C. pipiens*, there is a significant metabolic shift characterized by the downregulation of genes related to digestive enzymes, including trypsin and a chymotrypsin-like protease (Robich and Denlinger, 2005). Proteases can affect fatty acid accumulation, vital for energy during dormancy. For instance, cathepsin L is key in regulating lipid storage; silencing its gene leads to reduced lipid accumulation (Chen J. et al., 2022). Elastase, an enzyme crucial for protein digestion and remodeling, shows varying activity during diapause, suggesting that proteolytic activity is vital for the survival and subsequent emergence of the diapausing insect (Zaobidna et al., 2014). A comparison of diapausing and non-diapausing insects revealed significant changes in the expression of proteins related to metabolism and cellular protection. These changes include the regulation of heat-shock proteins and enzymes involved in protein synthesis and degradation, indicating that proteolytic enzymes are crucial for the diapause mechanism and the maintenance of cellular integrity during this period (Chen et al., 2010). Heat shock increases are often linked to diapause preparation (Denlinger, 2002). Heat shock proteins play a crucial role in maintaining cell homeostasis, particularly in stress response, by interacting with substrate proteins. Their expression is induced under stress conditions, even when protein synthesis is inhibited. During diapause the regulation of heat shock protein genes occurs differently, allowing for continued protein production without general disruption (Rinehart et al., 2007; King and MacRae, 2015). Regarding enzymatic regulation, in diapausing strains of the spongy moth, *Lymantria dispar*, proteolytic enzymes like trypsin and chymotrypsin activity is lower than in non-diapausing strains. This suggests that the suppression of proteolytic activity may be a key factor in maintaining diapause, as enzyme activity is closely linked to the insect’s developmental processes (Lee et al., 1997). One of the key enzymes involved in diapause is diapause-associated protein kinase (DAPK), which is responsible for phosphorylating and activating various proteins involved in diapause regulation. DAPK activity is regulated by phosphorylation and dephosphorylation events, which are influenced by the hormonal signals mentioned earlier. When the body experiences low energy levels due to factors like hypoxia and metabolic stresses, AMP-activated protein kinase (AMPK) is activated. This helps to turn on alternative pathways for producing ATP, ensuring enough energy to support the organism as overall energy production decreases during diapause. AMPK regulates cellular functions by phosphorylating metabolic enzymes and protein synthesis factors (Lu and Xu, 2010; MacRae, 2010; Gu et al., 2020).

**TABLE 9:** Proteolytic enzymes and their function in insect diapause.

Regulatory enzyme	Function	References
Trypsin	A serine protease cleaves long-chain protein molecules into smaller fragments	Rawlings and Barrett (1994)
Chymotrypsin	Hydrolyzes the peptide bonds of tryptophan, leucine, tyrosine, and phenylalanine residues	Cotten (2020a)
Cathepsin	A lysosomal cysteine proteinase that catalyzes the protein catabolism	Falke et al. (2024)
Elastase	A serine protease cleavages carboxyl groups on small hydrophobic amino acids, including glycine, alanine, and valine	Cotten (2020b)
Protein kinase	Catalyzes the transfer of a phosphate group from ATP to the serine or threonine residues of target proteins	Devang et al. (2021)

#### Hormonal manipulation to disrupt diapause in pest species

Hormonal manipulation through juvenile hormone analogs, inhibitors, and neuropeptide interventions is an effective method for disrupting diapause in various pest species, offering new strategies for population management. In *Galeruca daurica*, the application of the juvenile hormone analog methoprene at the pre-diapause stage delayed diapause onset, inhibited lipid accumulation, and altered key metabolic gene expression, preventing adults from entering diapause (Ma et al., 2021). For *Aedes albopictus*, the insect growth regulator pyriproxyfen effectively terminated embryonic diapause when applied to newly deposited and fully embryonated eggs, resulting in high termination rates and significant mortality, which is promising for controlling overwintering mosquitoes (Suman et al., 2015). Research on *Helicoverpa zea* showed that agonists mimicking diapause hormone can terminate or prevent pupal diapause when given during the larval stage, while antagonists inhibit this termination. This technique can disrupt pest populations by forcing insects to remain active in unfavorable conditions, reducing their survival and reproduction (Zhang et al., 2011). Overall, hormonal manipulation offers a powerful tool for targeting diapause to enhance pest management strategies.

### Conclusion

Enzyme-hormone interactions during diapause are essential for insect survival in adverse conditions. These interactions regulate the conversion and activation of ecdysteroids, preventing premature development. Additionally, they maintain the balance of juvenile hormone levels by managing the synthesis and degradation of relevant enzymes, thereby suppressing signals for growth and reproduction. Metabolic enzymes are also activated to produce cryoprotectants while keeping metabolic activity low. Together, these mechanisms create a stable physiological state that enables insects to endure until environmental cues indicate the appropriate time to resume development. Future research should aim to unravel the complex molecular interactions between hormones and enzymes. This understanding will enhance our knowledge of insect biology and guide the creation of innovative pest management strategies, as well as efforts to protect beneficial insect species. By manipulating the breakdown of juvenile hormones using inhibitors or analogs, we can disrupt the reproductive diapause of agricultural pests, making them more susceptible to adverse environmental conditions. Juvenile hormone esterase inhibitors can extend diapause in beneficial insects, such as pollinators, which helps them survive unseasonable climate changes. While diapause enhances resilience and can prolong lifespan, extended periods of dormancy pose significant risks for both individual insects and entire populations. One major concern is the reduction in reproductive capacity; prolonged dormancy can delay reproduction, resulting in fewer offspring when conditions eventually improve. Additionally, diapause is typically synchronized with the life cycles of host pests. If diapause lasts too long, it may lead to a mismatch between the emergence of beneficial insects and the availability of their prey or hosts, potentially reducing their effectiveness in controlling pests (Hoy, 1978; Bean et al., 2007; Meuti et al., 2024). Genetically engineering crop systems to disrupt hormone signaling specific to diapause could selectively target pest populations while preserving non-diapause species. Additionally, gaining insights into the synthesis of trehalose and glycerol could enhance cryopreservation techniques, aiding in the preservation of biological materials like pollinators and biological control agents. Inhibiting prothoracicotropic hormone or ecdysteroid production in overwintering pests may prolong diapause by delaying its termination and preventing reproduction. Disrupting the enzymes involved in cryoprotectant synthesis, particularly aldose reductase, which is essential for glycerol production, can make pest insects less tolerant to freezing temperatures. This may lead to higher mortality rates during their diapause phase. Conversely, by enhancing the cryoprotectant pathways in beneficial insects through genetic or environmental interventions, we could increase their resilience to climate variability. This, in turn, would enhance their contributions to the ecosystem. Targeting enzymes related to glycolysis during diapause may cause energy depletion in pests, which can ultimately decrease their survival rates during winter. Additionally, monitoring environmental factors like photoperiod and temperature that affect hormonal and enzymatic pathways during diapause can improve pest forecasting models. Future research directions may include the following suggestions: Investigating CRISPR-based techniques for modifying diapause-related genes to enhance pest management and conservation efforts; Exploring the interactions between hormones, such as juvenile hormone, prothoracicotropic hormone, and insulin-like peptides, to identify new strategies for regulating diapause, and examining diapause in the context of extreme environmental conditions, such as drought or heat, to develop a comprehensive framework for climate adaptation.
